# Circadian BMAL1 regulates mandibular condyle development by hedgehog pathway

**DOI:** 10.1111/cpr.12727

**Published:** 2019-11-20

**Authors:** Shaoling Yu, Qingming Tang, Mengru Xie, Xin Zhou, Yanlin Long, Yanling Xie, Fengyuan Guo, Lili Chen

**Affiliations:** ^1^ Department of Stomatology Union Hospital Tongji Medical College Huazhong University of Science and Technology Wuhan China

**Keywords:** BMAL1, chondrogenesis and endochondral ossification, genome‐wide RNA sequencing, patched homologue 1 (PTCH1), prepuberty and early puberty periods

## Abstract

**Objective:**

Chondrogenesis and endochondral ossification in mandibular condyle play crucial roles in maxillofacial morphogenesis and function. Circadian regulator brain and muscle arnt‐like 1 (BMAL1) is proven to be essential for embryonic and postnatal development. The goal of this study was to define the functions of BMAL1 in the embryonic and postnatal growth of mandibular condylar cartilages (MCC).

**Materials and Methods:**

Micro‐CT, TUNEL staining and EdU assay were performed using BMAL1‐deficient mice model, and in vitro experiments were performed using rat chondrocytes isolated from MCC. RNA sequencing in mandibular condyle tissues from *Bmal1* ^‐/‐^ mice and the age‐matched wild‐type mice was used for transcriptional profiling at different postnatal stages.

**Results:**

The expression levels of BMAL1 decrease gradually in MCC. BMAL1 is proved to regulate sequential chondrocyte differentiation, and its deficiency can result in the impairment of endochondral ossification of MCC. RNA sequencing reveals hedgehog signalling pathway is the potential target of BMAL1. BMAL1 regulates hedgehog signalling and affects its downstream cascades through directly binding to the promoters of *Ptch1* and *Ihh*, modulating targets of hedgehog signalling which is indispensable for endochondral ossification. Importantly, the short stature phenotypes caused by BMAL1 deficiency can be rescued by hedgehog signalling activator.

**Conclusions:**

Collectively, these results indicate that BMAL1 plays critical roles on chondrogenesis and endochondral ossification of MCC, giving a new insight on potential therapeutic strategies for facial dysmorphism.

## 1 INTRODUCTION

Mandibular condyle plays a pivotal role in the development of oro‐facial complex, since it functions as a regional adaptive growth site.^1^ Mandibular condylar cartilage (MCC) caps the surface of the mandibular condyle and serves as a site of articulation with the mandible, as well as a locus for chondrogenesis and endochondral ossification that contributes to morphogenesis and growth of the mandible in length and height.^2^ Unlike other articular cartilage within limb joints, MCC is considered as a secondary cartilage and its growth is categorized into two phases, maturation and mineralization.^3^, ^4^ Deviations in the growth of MCC affect the function of occlusion, leading to severe facial dysmorphism.^5^

The growth process of MCC is not only strictly regulated by a variety of local autocrine/paracrine factors including intermittent parathyroid hormone (I‐PTH) and insulin‐like growth factors (IGFs), but also under the control of complex transcriptional networks, such as transforming growth factor β (TGF‐β)/bone morphogenic protein (BMP) and circadian clock transcription outputs.^1^, ^6^, ^7^ The circadian clock is an endogenous rhythm and is of considerable interest due to its potential effects on tissue growth and structural development.^8^, ^9^ The transcription factor brain and muscle arnt‐like 1 (BMAL1) is the core driver of the circadian pacemaking in mammals and orchestrates organisms to adapt to the 24‐hours light‐dark cycle.^10^, ^11^, ^12^ Accumulating evidences suggest that BMAL1 plays pivotal roles in embryonic and postnatal development, including oocyte fertilization, follicle development, angiogenesis and neurodevelopment.^13^, ^14^, ^15^, ^16^ Our recent study has indicated that BMAL1 controls osteogenic differentiation and osteoclast differentiation in mandible.^17^ However, the roles of BMAL1 in chondrogenesis and endochondral ossification of mandibular condyle and its molecular mechanism are not been explained clearly.

In this study, we have verified that BMAL1 plays vital roles in chondrogenesis and endochondral ossification of mandibular. Our findings provide new insights into the pathogenesis of deformities in MCC and present potential therapeutic strategies for facial dysmorphism.

## 2 MATERIALS AND METHODS

### 2.1 Animals

Homozygous BMAL1‐deficiency (*Bmal1* ^‐/‐^, B6.129‐*Arntl* ^tm1Bra^/J mice) mice^18^ were produced by breeding heterozygous BMAL1‐deficienct mating pairs (*Bmal1* ^+/–^) which were obtained originally from the Jackson Laboratory and kindly provided by Dr Ying Xu (Soochow University, China). *Bmal1* ^fl/fl^ mice were also obtained from Dr Ying Xu, and *Twist2*‐Cre mice were purchased from the Jackson Laboratories (No. 008712). All experimental mice were maintained on a C57BL/6J genetic background. All procedures were performed according to the ethical guidelines and approved by the Institutional Animal Care and Use Committee of Tongji Medical College (IACUC number: S739).

### 2.2 Micro‐CT

The mandibles of experimental mice were harvested and fixed. Scanning was performed at a voxel size of 9 μm by Micro‐CT (SkyScan 1176, Broker). The images were used to reconstruct the tomography scans and quantify. Bone mass was evaluated according to bone mineral density (BMD), bone volume/total volume (BV/TV), trabecular thickness (Tb.Th), bone surface area/bone volume (BS/BV), trabecular number (Tb.N) and trabecular separation (Tb.Sp).

### 2.3 RNA sequencing

Total RNA was extracted from distally mandibular condyle tissues of *Bmal1^‐/‐^* mice and wild‐type mice (each 3 pairs of 4‐, 6‐, 8‐ and 10‐week‐old). The samples were sent to BGI Tech. for quantification, RNA‐Seq library construction, sequencing and bioinformatics analysis (Appendix S1).

### 2.4 Other methods

Alcian blue and alizarin red staining, Edu staining assay, etc, were performed. Please see the Appendix S1 for more details.

### 2.5 Statistical analysis

Unless otherwise stated, all data were shown as mean ± standard deviation. The GraphPad Prism 7.0 software was used for statistical analysis. Statistically significant differences were determined by Student's *t* test or ANOVA. A *P*‐value <.05 was considered with statistical significance.

## 3 RESULTS

### 3.1 Circadian regulator BMAL1 decreases gradually in the development of MCC

**Figure 1:**
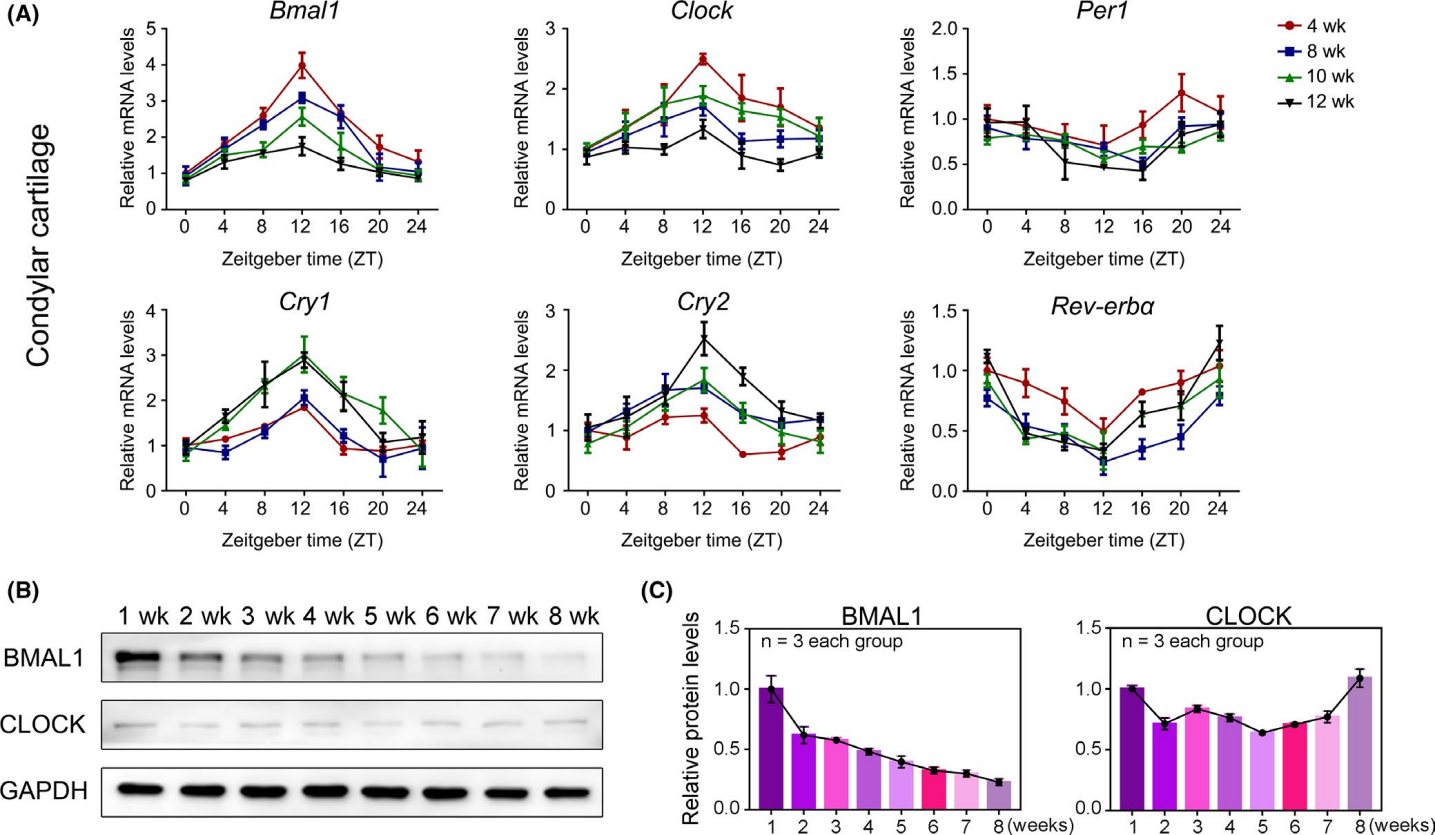
Circadian regulator BMAL1 is closely correlated with the development of MCC. A, qRT‐PCR analysis showed the mRNA levels of *Bmal1, Per1, Clock, Cry1, Cry2 and Rev‐erbα* at indicated time in cartilages of 4‐, 8‐, 10‐ and 12‐week‐old mice (n = 3 for each bar). B, C, Western blot analysis showed the protein levels of BMAL1 and CLOCK in the mandibular condyle cartilages at Zeitgeber time 10 (ZT10) (n = 3 independent experiments)

**Figure 2:**
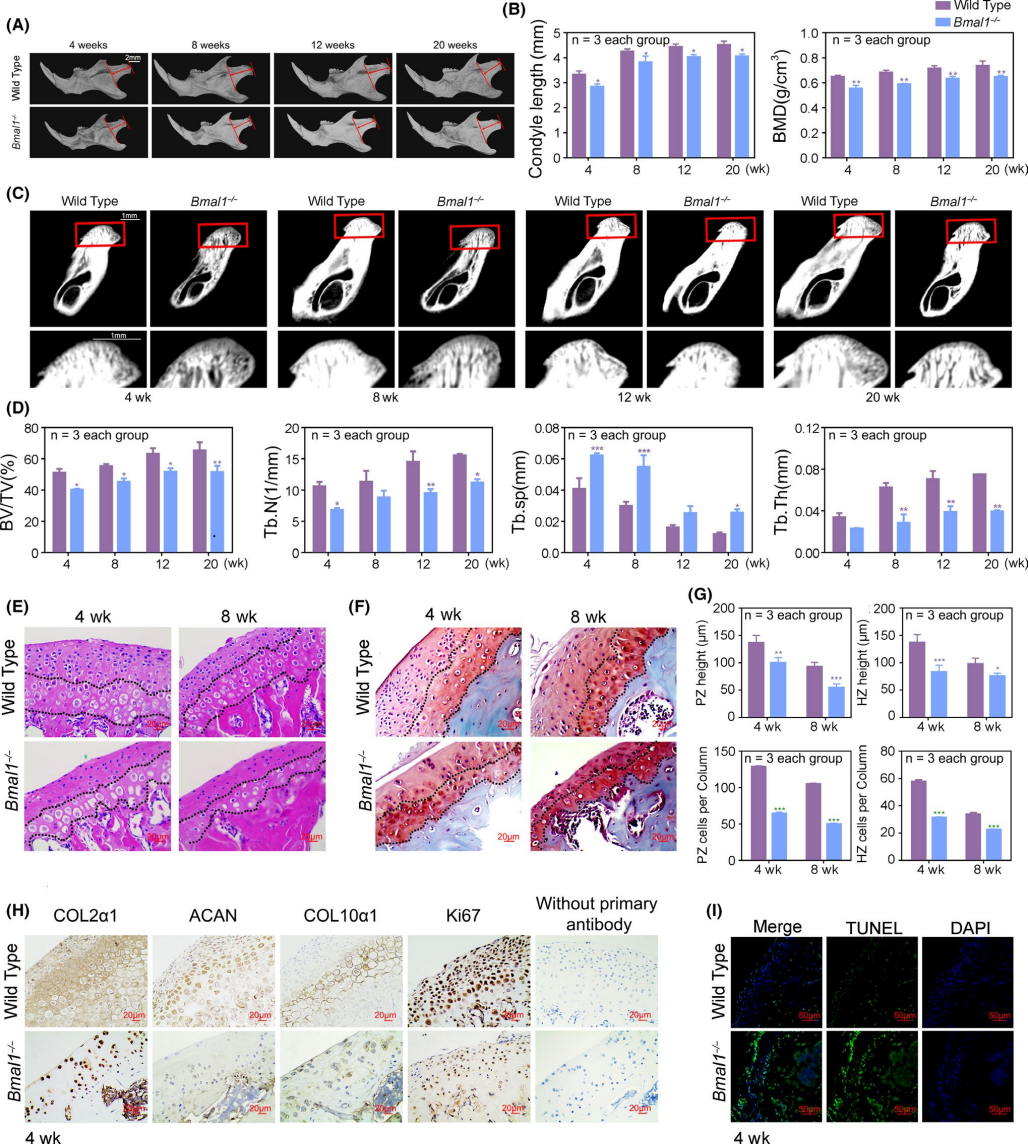
Loss of BMAL1 delays and reduces chondrogenesis and endochondral ossification in mandibular condyle. A, Representative images of micro‐CT reconstruction of mandibles (n = 3 per group). Red arrow indicated the mandibular condyle length. Scale bar, 2 mm. B‐D, Representative images and analysis of length, BMD, BV/TV, Tb.N, Tb.sp and Tb.Th of mandibular condyles (n = 3 for each bar). Scale bar, 1 mm. Data represented as mean ± SD, ^*^ *P* < .05, ^**^ *P* < .01, ^***^ *P* < .001. E, F, Representative images of H&E and S‐O staining of mandibular condyles at 4‐wk‐old and 8‐wk‐old (n = 3 per group). Scale bar, 20 μm. G, The height and the number of chondrocytes per column in PZ and HZ of mandibular condyle cartilages (n = 3 for each bar). Data represented as mean ± SD, ^***^ *P* < .001. H, Immunohistochemistry of COL2α1, ACAN, COL10α1 and Ki67 in mandibular condyles at ZT10, without primary antibody as negative group (n = 3 per group). Scale bar, 20 μm. I, The cell apoptosis of mandibular condyles was detected by TUNEL staining (n = 3 per group). Scale bar, 50 μm

To determine the functions of circadian clock in the growth of MCC, we measured the expression patterns and levels of core clock genes in MCC from various week‐old mice. We revealed that the phases of circadian rhythms were consistent across different developmental stages (Figure 1A). Meanwhile, we detected that the expression levels of circadian regulator BMAL1 decreased gradually in MCC, rather than other circadian proteins, including circadian locomotor output cycles kaput (CLOCK), period homologue drosophila 1/2 (PER1/2), cryptochrome 1/2 (CRY1/2), reverse‐erythroblastosis virus alpha (REV‐ERBα) and RAR‐related orphan receptor alpha (RORα) in MCC tissues of (1‐8)‐week‐old mice (Figure 1B, 1 and Figure S1). Traditionally, the thicknesses of proliferative layer zone (PZ) and hypertrophic layer zone (HZ) in MCC is gradually decreased in wild‐type mice (Figure 2E‐G). These results suggested that circadian regulator BMAL1 is closely correlated with the development of MCC.

### 3.2 Loss of BMAL1 delays and reduces chondrogenesis and endochondral ossification in mandibular condyle

To further confirm the role of BMAL1 in development and growth of MCC, we constructed global BMAL1‐deficiency (*Bmal1* ^‐/‐^) mice (Figure S2A). Here, we observed that the chondrogenesis of *Bmal1* ^‐/‐^ mice was slower and lesser compared with wild‐type mice at E14.5, E16.5 and E18.5 stages (Figure S2B, C). Consistently, the mandibular condyles of *Bmal1* ^‐/‐^ mice were smaller and shorter in height than those of the age‐matched wild‐type mice (Figure 2A). Micro‐CT analysis showed that the bone mineral density (BMD), bone volume/total volume (BV/TV), trabecular thickness (Tb.Th) and trabecular number (Tb.N) of *Bmal1* ^‐/‐^ mice significantly decreased and trabecular separation (Tb.Sp) increased relative to those wild‐type mice in the mandibular condylar regions (Figure 2B‐D). Morphological evaluation of MCC further verified that the height of PZ and HZ was diminished obviously, and the number of chondrocytes per column in PZ was decreased significantly compared to the age‐matched wild‐type mice (Figure 2E‐G). The basic components of cartilage matrix type II collagen (COL2α1), aggrecan (ACAN) and type X collagen (COL10α1) were dramatically less in *Bmal1* ^‐/‐^ mice at prenatal and postnatal development stage (Figure 2H and Figure S2D). Immunohistochemical staining for Ki67 revealed that chondrocyte proliferation was significantly attenuated in PZ (Figure 2H and Figure S2E). Moreover, TUNEL staining showed that cell apoptosis of chondrocyte was increased in MCC of *Bmal1* ^‐/‐^ mice compared with the age‐matched wild‐type mice (Figure 2I and Figure S2F).

**Figure 3:**
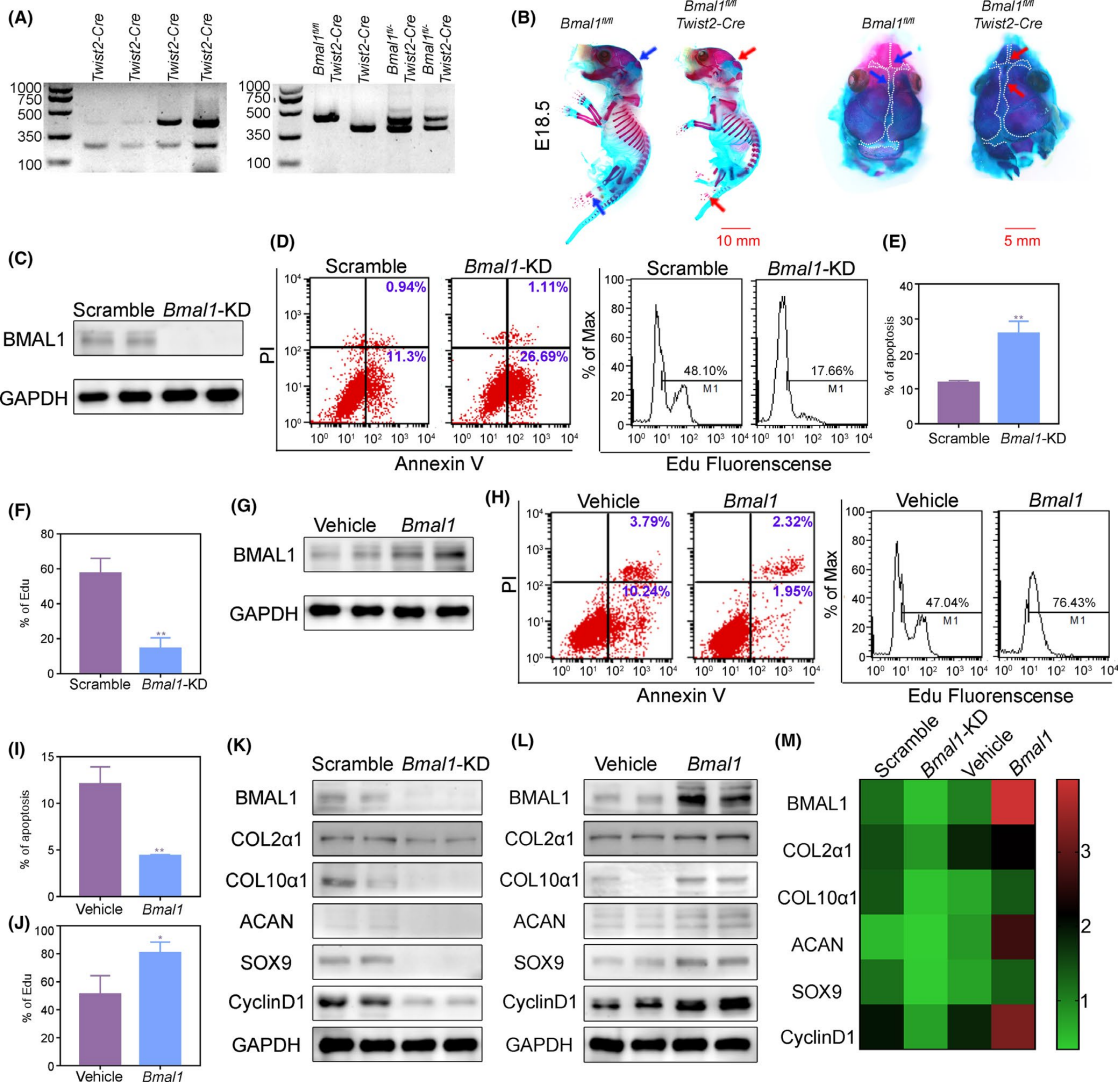
Knockdown of BMAL1 reduces proliferation and increases apoptosis in mandibular condylar chondrocytes. A, Genotyping identification of *Twist2‐Cre, Bmal1^fl/fl^Twist2‐Cre and Bmal1^fl/‐^Twist2‐Cre* mice by PCR. B, Alcian blue and Alizarin red staining of *Bmal1^fl/fl^Twist2‐Cre* or *Bmal1^fl/fl^* mice type embryos at E18.5 (n = 3 per group). Scar bar, 10 mm. C, BMAL1 knockdown was assessed by Western blot. D‐J, Flow cytometry analysis of cell proliferation and cell apoptosis in *Bmal1*‐knockdown or overexpressing chondrocytes (n = 3 for each bar). Data represented as mean ± SD. ^*^ *P* < .05, ^**^ *P* < .01. K‐M, Western blot analysis of relative protein levels in *Bmal1*‐knockdown or overexpressing chondrocytes. The heatmap (M) shows the analysis of Western blot

To test whether BMAL1 has intrinsic functionality in chondrocyte proliferation and cartilage matrix productions, we constructed conditional *Bmal1* knockout mice in mesenchymal stem cells (MSCs) (*Bmal1* ^fl/fl^; *Twist2*‐Cre) (Figure 3A). The results showed that the chondrogenesis and endochondral ossification of the conditional *Bmal1* knockout mice were significantly reduced, suggesting that BMAL1 in MSCs was closely associated with chondrogenesis and endochondral ossification (Figure 3B). To further verify the function of BMAL1 in chondrocytes, we extracted primary chondrocytes from mandibular condylar tissues of wild‐type rat and knocked down *Bmal1* using CRISPR/CAS9 system (Figure 3C). We found that BMAL1 deficiency obviously decreased cell proliferation and increased cell apoptosis in chondrocytes (Figure 3D‐F). The critical components of cartilage matrix COL2α1, ACAN and COL10α1, and the chondrocyte proliferation markers ex‐determining region Y (SRY)‐box 9 (SOX9) and CyclinD1, were all significantly downregulated in BMAL1‐deficient chondrocytes (Figure 3K, M). Consistently, BMAL1 overexpression yielded the opposite results in chondrocytes (Figure 3G‐J, L and M). Collectively, these findings verified that BMAL1 deficiency inhibits chondrogenesis and endochondral ossification in mandibular condyle by reducing sequential chondrocyte differentiation.

### 3.3 Along with the development of mandibular condyle, the transcriptional effects of BMAL1 in MCC are gradually decreased

**Figure 4:**
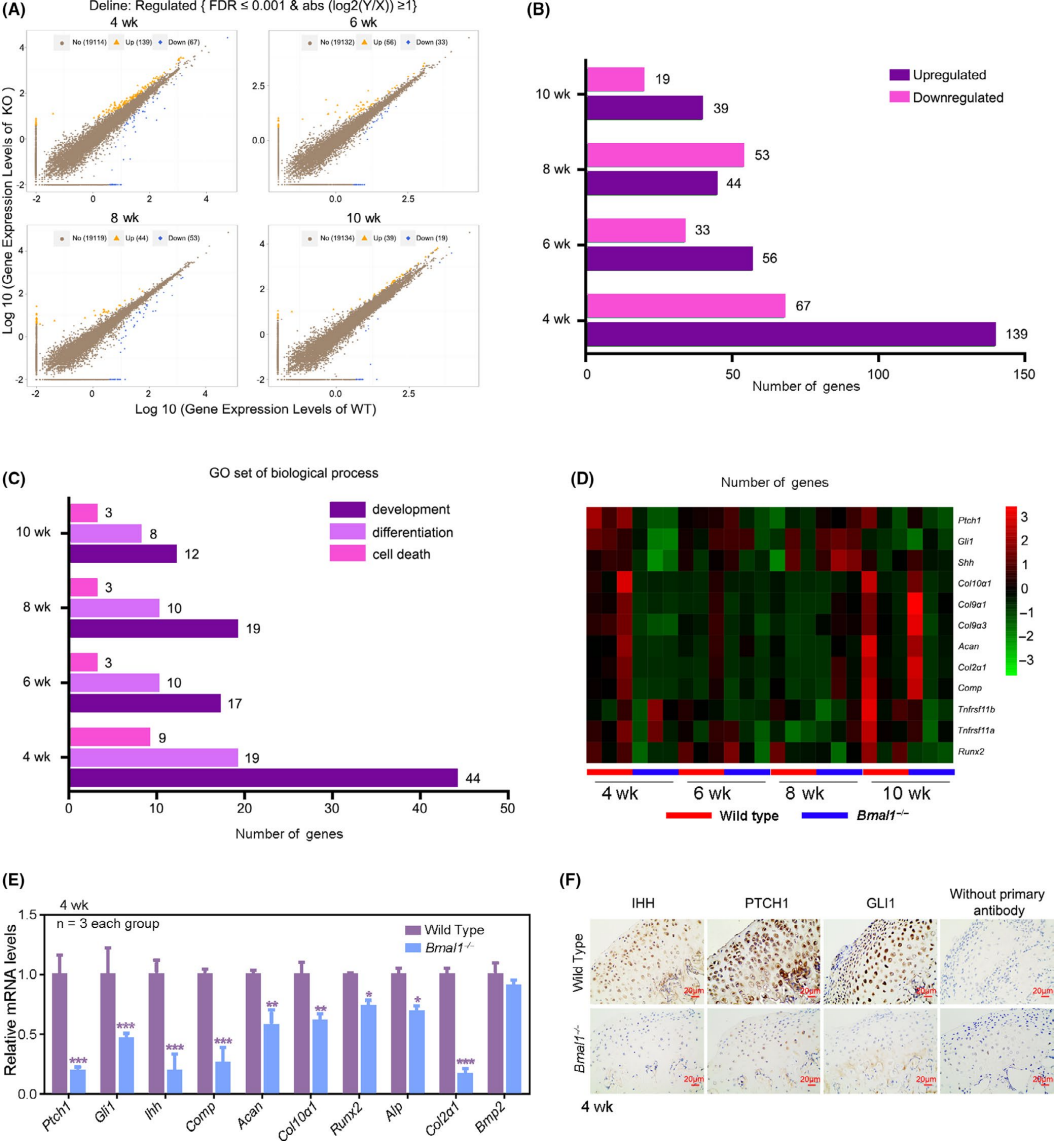
Along with the development of mandibular condyle, the transcriptional effects of BMAL1 in MCC are gradually decreased. A, Scatter plot of differentially expressed genes (DEGs) in *Bmal1^‐/‐^* and the age‐matched control wild‐type mice (n = 3 pairs per group). B, C, DEGs and associated Gene Ontology (GO) across 4, 6, 8 and 10 wk of *Bmal1^‐/‐^* and wild‐type mice. D, Heatmap showed some DEGs that were related to cartilage development. E, Confirmation of the DEGs in the MCC from 4‐wk‐old wild‐type and *Bmal1* ^‐/‐^ mice by qRT‐PCR analysis (n = 3 for each bar). Data represented as mean ± SD, ^*^ *P* < .05, ^**^ *P* < .01, ^***^ *P* < .001. F, Immunohistochemistry of IHH, PTCH1 and GLI1 according to these studies 45, 46, 47 in the MCC from 4‐week‐old wild‐type and *Bmal1* ^‐/‐^ mice (n = 3 per group), without primary antibody as negative group. Scale bar, 20 μm

BMAL1 is a widely acknowledged transcriptional activator.^19^ To determine the transcriptional effects of BMAL1 on the development of MCC, we performed a genome‐wide RNA sequencing to acquire the transcriptional profile in mandibular condyles from 4‐, 6‐, 8‐ and 10‐week‐old *Bmal1* ^‐/‐^ mice and the age‐matched wild‐type mice (Figure 4A). Similarity of transcriptional profiles among the three replicates in the same group was determined by a Pearson correlation coefficient (PCC) analysis. The three replicates showed high correlation with each other (Figure S3). Importantly, we found that the number of differentially expressed genes (DEGs) in the group of 4 weeks of age was the largest, with 206 differential genes, and the 10 weeks of age was the least, with 58 differential genes (Figure 4B). GO analysis revealed that many DEGs were enriched in development process, differentiation and cell death. Remarkably, the number of DEGs in development and differentiation during prepuberty and puberty (4 and 6 weeks of age) was almost twice as many as in young adulthood (8 and 10 weeks of age; Figure 4C). Then, we focused on the candidate gene set that related to chondrogenesis and endochondral ossification, including *Gli1*, *Col2α1* and *Col10α1*. We found that these genes had the similar spatio‐temporal expression patterns to *Bmal1*, with a higher expression level during prepuberty and puberty (Figure 4D). Moreover, the expression changes of these genes after knocking down *Bmal1* were greater during prepuberty and puberty than young adulthood (Figure 4D). These results verified that the transcriptional effects of BMAL1 in MCC are gradually decreased which is along with the development of mandibular condyle.

### 3.4 Hedgehog signalling is under the strict control of BMAL1 and is critical for endochondral ossification

Due to the higher expression levels of *Bmal1* and the genes related to chondrogenesis and endochondral ossification in prepuberty and puberty, the data of RNA sequencing from 4‐week‐old mice were analysed and further verified by quantitative PCR and immumohistochemical staining. We found that *Ihh*, *Ptch1* and *Gli1* were significantly downregulated along with the genes related to chondrogenesis and endochondral ossification (Figure 4D‐F), suggesting that hedgehog (Hh) pathway was under the strict control of BMAL1 in mandibular condyles. Hedgehog pathway plays critical roles in sequential chondrocyte differentiation.^20^ To verify the effects of Hh signalling in chondrocytes from mandibular condyle, we used Hh signalling activator smoothened agonist (SAG; 0.1, 0.5 or 1.0 μmol/L) or inhibitor vismodegib (GDC‐0449; 0.1, 0.5 or 1.0 μmol/L). We observed increased proliferation and decreased apoptosis after SAG supplement (Figure S4A‐D). In contrast, GDC‐0449 resulted in significant decrease of cell proliferation and increase of cell apoptosis (Figure S4A‐D). Western blot assay showed that the expression levels of indian hedgehog (IHH), patched homologue 1 (PTCH1), GLI‐Kruppel family member (GLI1), COL2α1, ACAN, COL10α1, matrix metallopeptidase 13 (MMP13), cell apoptosis regulators apoptosis regulator Bcl‐2 (BCL2) and bcl‐2‐like protein 1 (BCL‐XL), chondrocyte proliferation markers SOX9 and CyclinD1 increased with SAG supplement (Figure S4E, F), while the expression levels of all above genes decreased with GDC‐0449 treatment (Figure S4G, H). These results suggested that the loss of BMAL1 may decrease chondrocyte proliferation and cartilage matrix production by downregulating Hh signalling.

### 3.5 BMAL1 regulates sequential chondrocyte differentiation through directly activating Ptch1 and Ihh transcription

**Figure 5:**
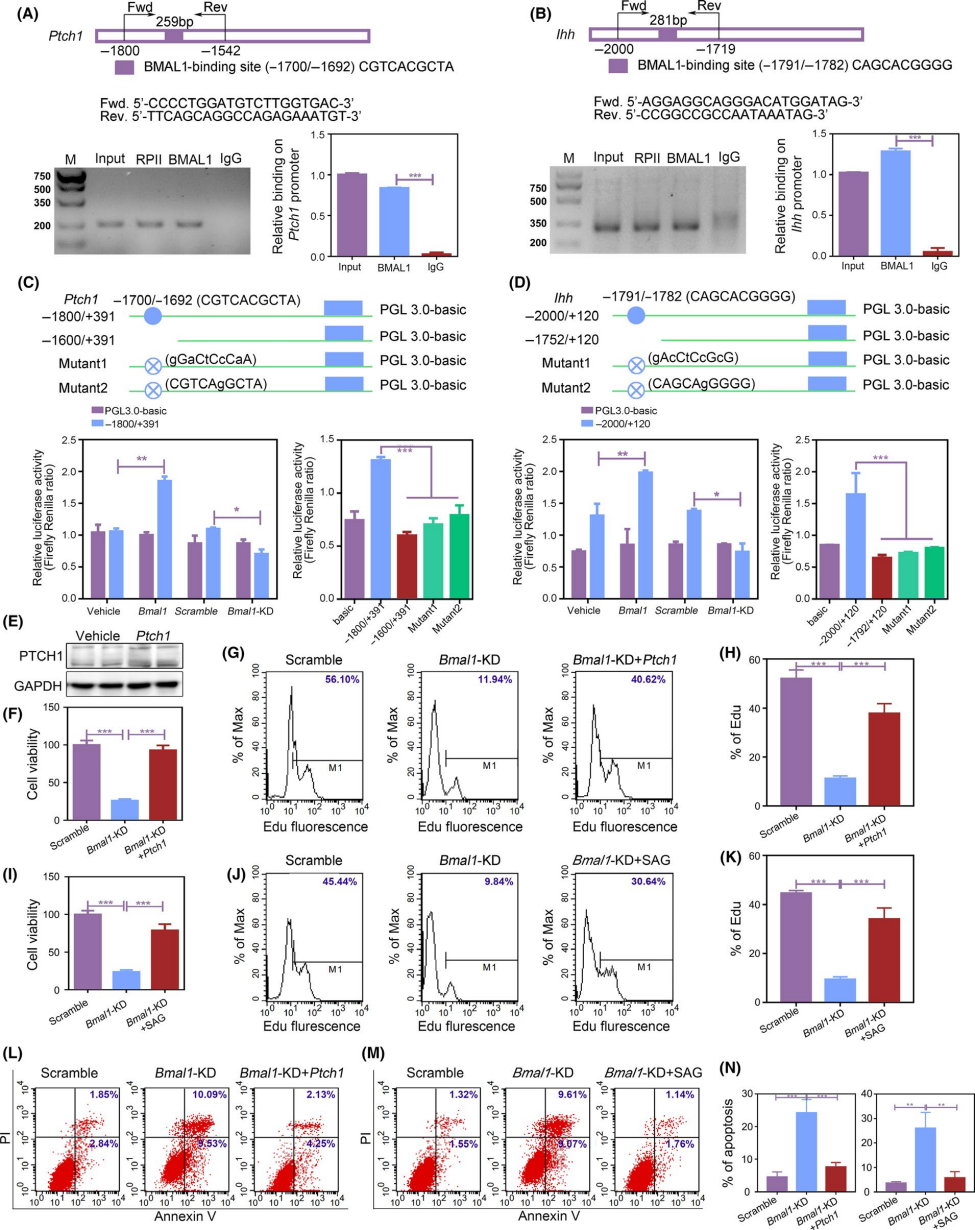
BMAL1 regulates sequential chondrocyte differentiation through directly activating *Ptch1* and *Ihh* transcription. A, B, The transcription factor BMAL1 bound to the *Ptch1* and *Ihh* in primary mandibular condylar chondrocytes. Chromatin immunoprecipitation assays were performed using anti‐BMAL1 with anti‐IgG as a negative control (n = 3 for each bar). Data represented as mean ± SEM. ^***^ *P* < .001. C, D, Dual‐luciferase assays were performed to measure the activities of *Ptch1* and *Ihh* promoters in primary mandibular condylar chondrocytes. Filled blue circle, BMAL1‐binding site. Filled crossed blue circle, mutant of BMAL1‐binding site (n = 3 for each bar). Data represented as mean ± SEM. ^*^ *P* < .05, ^**^ *P* < .01, ^***^ *P* < .001. E, PTCH1 overexpression was assessed by Western blot. F‐N, The cell proliferation and cell apoptosis were detected by CCK‐8 (F and I), EdU staining (G, H, I, J) and flow cytometry assays (L‐N) in BMAL1 knockdown with PTCH1 overexpressing or SAG supplemented primary mandibular condylar chondrocytes (n = 3 for each bar). Data represented as mean ± SD. ^***^ *P* < .001

As a classical transcription activator, BMAL1 plays its roles by combining the E‐box site in the promoter of downstream target genes and activating their transcription.^11^ To further clarify the underlying mechanisms in which BMAL1 controls Hh signalling, the ChIP assays were employed to examine BMAL1‐binding site within *Ptch1* and *Ihh* promoter regions. Our results indicated that BMAL1 selectively bound to the promoter (−1800/‐1542, 259 bp) of *Ptch1* (Figure 5A). We also found that BMAL1 could bind to *Ihh* promoter region which was identical with the similar report 21 (Figure 5B). Dual‐luciferase assay further verified that BMAL1 improved the activity of *Ptch1* and *Ihh* promoters, but not those of the mutants with BMAL1‐binding site being either deleted or mutated. In addition, we found that BMAL1 overexpression could further improve the activity of both *Ptch1* and *Ihh* promoters (Figure 5C, 5).

To verify the necessary function of PTCH1 in BMAL1‐regulated chondrogenesis and endochondral ossification, we explored whether PTCH1 overexpression or Hh signalling activator SAG could rescue the phenotype caused by BMAL1 deficiency in chondrocytes. The results indicated that PTCH1 overexpression or SAG reversed the reduction of chondrocyte proliferation and cartilage matrix production and the increase of chondrocyte apoptosis induced by BMAL1 knockdown (Figure 5E‐N and Figure S5). Taken together, these findings indicated that the loss of BMAL1 inhibits sequential chondrocyte differentiation through directly activating *Ptch1* and *Ihh* transcription.

### 3.6 Phenotypes caused by BMAL1 deficiency can be rescued by Hh signalling activator during prepuberty and early puberty periods

**Figure 6:**
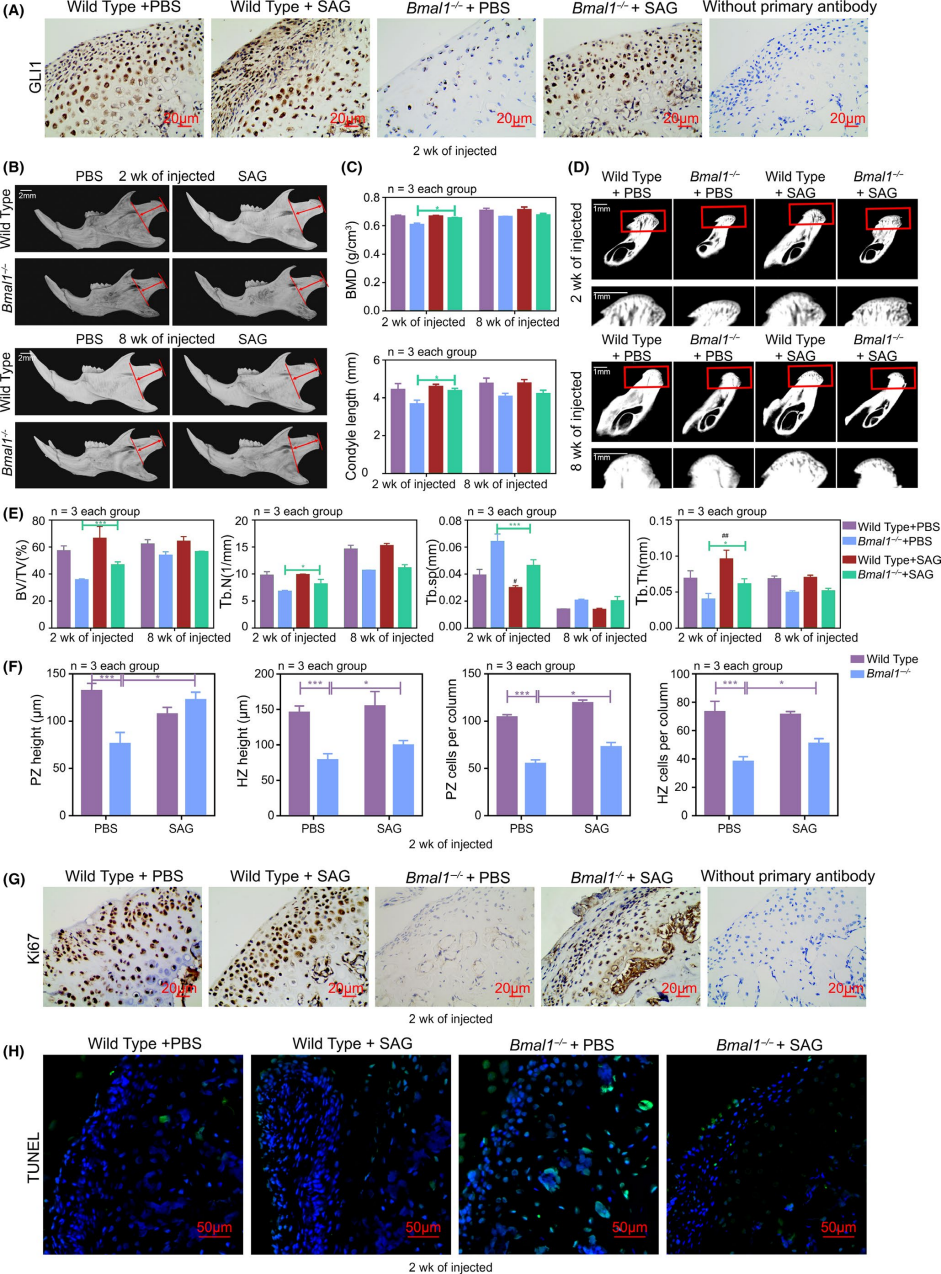
Phenotypes caused by BMAL1 deficiency are rescued by Hh signalling activator during prepuberty and early puberty periods. A, Immunohistochemistry of GLI1 in mandibular condyles at ZT10, without primary antibody as negative group. Scale bar, 20 μm. B, Images of micro‐CT reconstruction of the mandibles of wild‐type and *Bmal1^‐/‐^* mice with or without injection of SAG at 2 wk or 8 wk, 20 μg/g, three times a week for 4 wk (n = 3 per group). Red arrow indicated the mandibular condyle length. Scale bar, 2 mm. C‐E, Representative images and analysis of length, BMD, BV/TV, Tb.N, Tb.sp and Tb.Th of mandibular condyles (n = 3 for each bar). Scale bar, 1 mm. Data represented as mean ± SD, ^*^ *P* < .05, ^**^ *P* < .01. F, The height and the number of chondrocytes per column in PZ and HZ of mandibular condyle cartilages (n = 3 for each bar). Data represented as mean ± SD, ^***^ *P* < .001. G and H, The cell proliferation and cell apoptosis in mandibular condyles were detected by Ki67 and TUNEL staining at ZT10 (n = 3 per group). Scale bar, 20 μm, 50 μm

To determine whether activating Hh signalling can rescue the short malformation phenotypes of mandible caused by BMAL1 deficiency, SAG was injected intraperitoneally into *Bmal1* ^‐/‐^ mice during the prepuberty and early puberty periods (2 weeks of age) and young adulthood (8 weeks of age), respectively (20μg/g, three times a week, 4 weeks). Immunohistochemical staining for GLI1 demonstrated significant activation of the Hh pathway as defined by increased expression of GLI1 after the application of SAG (Figure 6A). Micro‐CT results showed that skeletal development in the puberty‐SAG‐injected *Bmal1* ^‐/‐^ mice was substantially ameliorative (Figure 6B‐E). H&E staining showed a significant increase in the number of PZ chondrocytes (Figure 6F and Figure S6A). Furthermore, there were more Ki67‐positive chondrocytes in PZ and less TUNEL‐positive chondrocytes in MCC from the *Bmal1* ^‐/‐^ mice at puberty‐SAG‐injected (Figure 6G, H). Consistently, the basic components of cartilage matrix COL2α1 were significantly increased after injecting SAG (Figure S6C). However, young adulthood‐SAG‐injected *Bmal1* ^‐/‐^ mice did not response effectively (Figure 6B‐E and Figure S6B). Taken together, our results revealed that Hh signalling activator plays potential roles in improving bone mass reduction caused by BMAL1 deficiency, which was effective in prepuberty and early puberty periods intervention, rather than young adulthood treatment. These findings provide a new strategy for the clinical treatment of facial dysmorphism.

## 4 DISCUSSION

BMAL1 controls limb bone and knee cartilage homeostasis and maintains structural integrity, and its aberrant expression leads to osteopenia and predisposes knee cartilage to osteoarthritis‐like damage.^21^, ^22^ William found that BMAL1‐deficient mice display an earlier closure of growth plates than wild‐type mice.^23^ Takarada et al also found the body length measured from nose to anus and the longitudinal lengths of both tibia and femur decreased remarkably in BMAL1‐deficient mice than wild‐type mice.^21^ Our previous study also demonstrated that osteogenic differentiation is reduced in limb and mandible from *Bmal1* ^‐/‐^ mice.^17^ Unlike the limb bone and maxillary and mandibular body which mainly rely on intramembranous osteogenesis, MCC is considered as a secondary cartilage and has significant structural differences from primary cartilages.^24^ In our study, we found the body size was apparently smaller in BMAL1‐deficient mice than that in wild‐type mice as reported previously.^21^, ^25^, ^26^ Importantly, we observed that foetuses in BMAL1‐deficient mice appear insufficient cranial cartilage calcification, and smaller and short mandibular condyle phenotypes.

Although the effects of BMAL1 on osteoblast differentiation and endochondral ossification of growth plate have been well established, its roles and the underlying mechanism in the regulation of chondrogenesis and endochondral ossification in MCC have not been explained explicitly. Here, we identified BMAL1 constitutively expressed in chondrocytes controls the sequential chondrocyte differentiation process in MCC through a mechanism related to regulation of the transactivation of *Ptch1* and *Ihh*.^21^ IHH and PTCH1 are the core component of Hh pathway.^27^, ^28^, ^29^ Hh signalling activation is relied on the coupling of ligand IHH and receptor PTCH1.^30^, ^31^ Our results reveal the molecular mechanism in which BMAL1 can modulate double targets of Hh signalling which is indispensable for endochondral ossification in mandibular condyle. Recent studies have also verified that chondrocytes can directly transform into bone cells.^4^, ^32^ However, our RNA‐seq did not show a significance in transdifferentiation‐related molecules.

In this report, we verified that PTCH1 overexpression partially rescues Hh signalling in BMAL1‐deficient chondrocytes. PTCH1 is essential for activating Hh downstream signalling, and Hh activation depends on the binding of hedgehog and PTCH1. Unbound PTCH1 will suppress Hh signalling in turn.^33^, ^34^ When Hh is sufficient, PTCH1 mild overexpression can recruit much more Hh from the extracellular matrix and then activate Hh signalling greatly.^35^, ^36^ Besides, the effect of PTCH1 also has tissue specificity, which might explain the reason that the mild overexpression of PTCH1 results in the “rescue” effect in BMAL1‐deficient chondrocytes in this work.^37^, ^38^ As we know, SAG cannot directly increase the expression of IHH. According to literature, the transcription factor Gli1 in hedgehog pathway enhances the direct induction of both *Runx2* and *Runx3* transcription and RUNX2 can subsequently regulate chondrocyte maturation and proliferation through the induction of *Ihh* expression.^39^, ^40^, ^41^ This might be the reason that SAG increases *Ihh* expression though GLI1‐RUNX2‐*Ihh* signal axis in chondrocytes.

Orthopedic or orthodontic operation is the main clinical strategy to remedy facial dysmorphism.^42^ But, the operation is complicated and the side effects are serious. Due to its complex aetiology, it is indeed difficult to develop an effective strategy to prevent the deformities. SAG, a specific Hh signalling activator, is reported to enhance bone healing in a bone defect model.^43^ Supplemental SAG can also promote neural regeneration after ischaemic brain injury.^44^ In our in vivo experiment, we found that SAG injection significantly improves mandibular bone mass reduction caused by *Bmal1* knockout at prepuberty and early puberty periods rather than young adulthood, suggesting that SAG may be the potential candidates as novel therapeutic strategies for preventing the facial dysmorphism.

Collectively, our data demonstrate chondrogenesis and endochondral ossification in MCC are under the strict regulation of BMAL1 through a mechanism directly relevant to modulate Hh signalling and provide insights into the mechanism of facial dysmorphism. Moreover, this study presents a ponderable reference for juveniles to correct their undesirable life styles.

## CONFLICT OF INTEREST

The authors claim no competing interest.

## AUTHOR CONTRIBUTIONS

LC conceived the study; SY, QT and LC involved in study design; SY and QT investigated the study; SY collected the data; SY, YL, YX, MX, XZ and FG analysed the data; SY and QT wrote the manuscript; LC supervised the study; and LC acquired funding.

## Data Availability

The data that support the findings of this study are available on request from the corresponding author.

## References

[1] Patil AS , Sable RB , Kothari RM . Role of insulin‐like growth factors (IGFs), their receptors and genetic regulation in the chondrogenesis and growth of the mandibular condylar cartilage. J Cell Physiol. 2012;227(5):1796‐1804. 21732349 10.1002/jcp.22905

[2] Jing J , Hinton RJ , Jing Y , Liu Y , Zhou X , Feng JQ . Osterix couples chondrogenesis and osteogenesis in post‐natal condylar growth. J Dent Res. 2014;93(10):1014‐1021. 25192899 10.1177/0022034514549379 PMC4212325

[3] Shen G , Darendeliler MA . The adaptive remodeling of condylar cartilage–‐a transition from chondrogenesis to osteogenesis. J Dent Res. 2005;84(8):691‐699. 16040724 10.1177/154405910508400802

[4] Jing Y , Zhou X , Han X , et al. Chondrocytes directly transform into bone cells in mandibular condyle growth. J Dent Res. 2015;94(12):1668‐1675. 26341973 10.1177/0022034515598135 PMC4681473

[5] Pirttiniemi P , Peltomaki T , Muller L , Luder HU . Abnormal mandibular growth and the condylar cartilage. Eur J Orthod. 2009;31(1):1‐11. 19164410 10.1093/ejo/cjn117

[6] Mh OB , Dutra EH , Lima A , Nanda R , Yadav S . PTH [1‐34] induced differentiation and mineralization of mandibular condylar cartilage. Sci Rep. 2017;7(1):3226. 28607469 10.1038/s41598-017-03428-y PMC5468307

[7] Xiao D , Wang R , Hu J , Quan H . Spatial and temporal expression of Smad signaling members during the development of mandibular condylar cartilage. Exp Ther Med. 2017;14(5):4967‐4971. 29201201 10.3892/etm.2017.5186 PMC5704254

[8] Kaneshi Y , Ohta H , Morioka K , et al. Influence of light exposure at nighttime on sleep development and body growth of preterm infants. Scientific Rep. 2016;6:21680. 10.1038/srep21680 PMC4753683 26877166

[9] Bell‐Pedersen D , Cassone VM , Earnest DJ , et al. Circadian rhythms from multiple oscillators: lessons from diverse organisms. Nat Rev Genet. 2005;6(7):544‐556. 15951747 10.1038/nrg1633 PMC2735866

[10] Marcheva B , Ramsey KM , Buhr ED , et al. Disruption of the clock components CLOCK and BMAL1 leads to hypoinsulinaemia and diabetes. Nature. 2010;466(7306):627‐631. 20562852 10.1038/nature09253 PMC2920067

[11] Gustafson CL , Parsley NC , Asimgil H , et al. A slow conformational switch in the BMAL1 transactivation domain modulates circadian rhythms. Mol Cell. 2017;66(4):447‐457.e447. 28506462 10.1016/j.molcel.2017.04.011 PMC5484534

[12] Tang Q , Cheng B , Xie M , et al. Circadian clock gene bmal1 inhibits tumorigenesis and increases paclitaxel sensitivity in tongue squamous cell carcinoma. Cancer Res. 2017;77(2):532‐544. 27821487 10.1158/0008-5472.CAN-16-1322

[13] Jensen LD , Cao Z , Nakamura M , et al. Opposing effects of circadian clock genes bmal1 and period2 in regulation of VEGF‐dependent angiogenesis in developing zebrafish. Cell Rep. 2012;2(2):231‐241. 22884368 10.1016/j.celrep.2012.07.005

[14] Powell WT , LaSalle JM . Epigenetic mechanisms in diurnal cycles of metabolism and neurodevelopment. Hum Mol Genet. 2015;24(R1):R1‐9. 26105183 10.1093/hmg/ddv234 PMC4683361

[15] Xu J , Li Y , Wang Y , Xu Y , Zhou C . Loss of Bmal1 decreases oocyte fertilization, early embryo development and implantation potential in female mice. Zygote (Cambridge, England). 2016;24(5):760‐767. 10.1017/S0967199416000083 27140828

[16] Zhang J , Liu J , Zhu K , et al. Effects of BMAL1‐SIRT1‐positive cycle on estrogen synthesis in human ovarian granulosa cells: an implicative role of BMAL1 in PCOS. Endocrine. 2016;53(2):574‐584. 27117143 10.1007/s12020-016-0961-2

[17] Zhao J , Zhou X , Tang Q , et al. BMAL1 deficiency contributes to mandibular dysplasia by upregulating MMP3. Stem Cell Reports. 2018;10(1):180‐195. 29276151 10.1016/j.stemcr.2017.11.017 PMC5768965

[18] Bunger MK , Wilsbacher LD , Moran SM , et al. Mop3 is an essential component of the master circadian pacemaker in mammals. Cell. 2000;103(7):1009‐1017. 11163178 10.1016/s0092-8674(00)00205-1 PMC3779439

[19] Takahashi JS . Transcriptional architecture of the mammalian circadian clock. Nature Rev Genet. 2017;18(3):164‐179. 27990019 10.1038/nrg.2016.150 PMC5501165

[20] Yang J , Andre P , Ye L , Yang YZ . The Hedgehog signalling pathway in bone formation. Int J Oral Sci. 2015;7(2):73‐79. 26023726 10.1038/ijos.2015.14 PMC4817553

[21] Takarada T , Kodama A , Hotta S , et al. Clock genes influence gene expression in growth plate and endochondral ossification in mice. J Biol Chem. 2012;287(43):36081‐36095. 22936800 10.1074/jbc.M112.408963 PMC3476276

[22] Dudek M , Gossan N , Yang N , et al. The chondrocyte clock gene Bmal1 controls cartilage homeostasis and integrity. J Clin Invest. 2016;126(1):365‐376. 26657859 10.1172/JCI82755 PMC4701559

[23] Samsa WE , Vasanji A , Midura RJ , Kondratov RV . Deficiency of circadian clock protein BMAL1 in mice results in a low bone mass phenotype. Bone. 2016;84:194‐203. 26789548 10.1016/j.bone.2016.01.006 PMC4755907

[24] Inoue H , Nebgen D , Veis A . Changes in phenotypic gene expression in rat mandibular condylar cartilage cells during long‐term culture. J Bone Miner Res. 1995;10(11):1691‐1697. 8592945 10.1002/jbmr.5650101111

[25] Shimba S , Ogawa T , Hitosugi S , et al. Deficient of a clock gene, brain and muscle Arnt‐like protein‐1 (BMAL1), induces dyslipidemia and ectopic fat formation. PLoS ONE. 2011;6(9):e25231. 21966465 10.1371/journal.pone.0025231 PMC3178629

[26] McDearmon EL , Patel KN , Ko CH , et al. Dissecting the functions of the mammalian clock protein BMAL1 by tissue‐specific rescue in mice. Science. 2006;314(5803):1304‐1308. 17124323 10.1126/science.1132430 PMC3756687

[27] Cai H , Liu A . Spop promotes skeletal development and homeostasis by positively regulating Ihh signaling. Proc Natl Acad Sci U S A. 2016;113(51):14751‐14756. 27930311 10.1073/pnas.1612520114 PMC5187670

[28] Suh JM , Gao X , McKay J , McKay R , Salo Z , Graff JM . Hedgehog signaling plays a conserved role in inhibiting fat formation. Cell Metab. 2006;3(1):25‐34. 16399502 10.1016/j.cmet.2005.11.012

[29] Kazmers NH , McKenzie JA , Shen TS , Long F , Silva MJ . Hedgehog signaling mediates woven bone formation and vascularization during stress fracture healing. Bone. 2015;81:524‐532. 26348666 10.1016/j.bone.2015.09.002 PMC4640972

[30] Yoshida M , Hata K , Takashima R , et al. The transcription factor Foxc1 is necessary for Ihh‐Gli2‐regulated endochondral ossification. Nat Commun. 2015;6:6653. 25808752 10.1038/ncomms7653

[31] Ehlen HW , Buelens LA , Vortkamp A . Hedgehog signaling in skeletal development. Birth Defects Res C Embryo Today. 2006;78(3):267‐279. 17061262 10.1002/bdrc.20076

[32] Yang G , Zhu L , Hou N , et al. Osteogenic fate of hypertrophic chondrocytes. Cell Res. 2014;24(10):1266‐1269. 25145361 10.1038/cr.2014.111 PMC4185343

[33] Sun ZH , Liu YH , Liu JD , et al. MeCP2 regulates PTCH1 expression through DNA methylation in rheumatoid arthritis. Inflammation. 2017;40(5):1497‐1508. 28573530 10.1007/s10753-017-0591-8

[34] Cohen M , Kicheva A , Ribeiro A , et al. Ptch1 and Gli regulate Shh signalling dynamics via multiple mechanisms. Nat Commun. 2015;6:6709. 25833741 10.1038/ncomms7709 PMC4396374

[35] Holtz AM , Peterson KA , Nishi Y , et al. Essential role for ligand‐dependent feedback antagonism of vertebrate hedgehog signaling by PTCH1, PTCH2 and HHIP1 during neural patterning. Development (Cambridge, England). 2013;140(16):3423‐3434. 10.1242/dev.095083 PMC3737722 23900540

[36] Chen Y , Struhl G . Dual roles for patched in sequestering and transducing Hedgehog. Cell. 1996;87(3):553‐563. 8898207 10.1016/s0092-8674(00)81374-4

[37] Chang H , Li Q , Moraes RC , Lewis MT , Hamel PA . Activation of Erk by sonic hedgehog independent of canonical hedgehog signalling. Int J Biochem Cell Biol. 2010;42(9):1462‐1471. 20451654 10.1016/j.biocel.2010.04.016 PMC3038129

[38] Monkkonen T , Landua JD , Visbal AP , Lewis MT . Epithelial and non‐epithelial Ptch1 play opposing roles to regulate proliferation and morphogenesis of the mouse mammary gland. Development (Cambridge, England). 2017;144(7):1317‐1327. 10.1242/dev.140434 PMC5399619 28275010

[39] Yoshida CA , Yamamoto H , Fujita T , et al. Runx2 and Runx3 are essential for chondrocyte maturation, and Runx2 regulates limb growth through induction of Indian hedgehog. Genes Dev. 2004;18(8):952‐963. 15107406 10.1101/gad.1174704 PMC395853

[40] Komori T . Regulation of proliferation, differentiation and functions of osteoblasts by Runx2. Int J Mol Sci. 2019;20(7):1694. 10.3390/ijms20071694 PMC6480215 30987410

[41] Kim EJ , Cho SW , Shin JO , Lee MJ , Kim KS , Jung HS . Ihh and Runx2/Runx3 signaling interact to coordinate early chondrogenesis: a mouse model. PLoS ONE. 2013;8(2):e55296. 23383321 10.1371/journal.pone.0055296 PMC3562241

[42] Chiu G , Chang C , Roberts WE . Interdisciplinary treatment for a compensated Class II partially edentulous malocclusion: orthodontic creation of a posterior implant site. Am J Orthod Dentofacial Orthop. 2018;153(3):422‐435. 29501118 10.1016/j.ajodo.2016.11.029

[43] Lee S , Wang C , Pan HC , et al. Combining smoothened agonist and NEL‐like protein‐1 enhances bone healing. Plast Reconstr Surg. 2017;139(6):1385‐1396. 28198775 10.1097/PRS.0000000000003367 PMC5443697

[44] Chechneva OV , Mayrhofer F , Daugherty DJ , et al. A Smoothened receptor agonist is neuroprotective and promotes regeneration after ischemic brain injury. Cell Death Dis. 2014;5:e1481. 25341035 10.1038/cddis.2014.446 PMC4649529

[45] Carbone A , Carballo C , Ma R , et al. Indian hedgehog signaling and the role of graft tension in tendon‐to‐bone healing: evaluation in a rat ACL reconstruction model. J Orthop Res. 2016;34(4):641‐649. 26447744 10.1002/jor.23066 PMC6345400

[46] Pang P , Shimo T , Takada H , et al. Expression pattern of sonic hedgehog signaling and calcitonin gene‐related peptide in the socket healing process after tooth extraction. Biochem Biophys Res Commun. 2015;467(1):21‐26. 26427874 10.1016/j.bbrc.2015.09.139

[47] Kureel J , John AA , Dixit M , Singh D . MicroRNA‐467g inhibits new bone regeneration by targeting Ihh/Runx‐2 signaling. Int J Biochem Cell Biol. 2017;85:35‐43. 28163186 10.1016/j.biocel.2017.01.018

