# Correction to “Circadian BMAL1 Regulates Mandibular Condyle Development by Hedgehog Pathway”

**DOI:** 10.1111/cpr.70111

**Published:** 2025-08-05

S. Yu, Q. Tang, M. Xie, et al., “Circadian BMAL1 Regulates Mandibular Condyle Development by Hedgehog Pathway,” *Cell Proliferation* 53, no. 1 (2020): e12727, https://doi.org/10.1111/cpr.12727.

The images of the immunohistochemistry assays for GLI1 in the “Wild Type + PBS” group (Figure 6A), Ki67 in “Wild Type + SAG” group (Figure 6G) and COL2α1 in “Wild Type + SAG” group (Figure S6C) were incorrect. The corrected versions of Figures 6A, 6G and S6C are provided below. These corrections do not alter any of the findings or conclusions reported in this article.

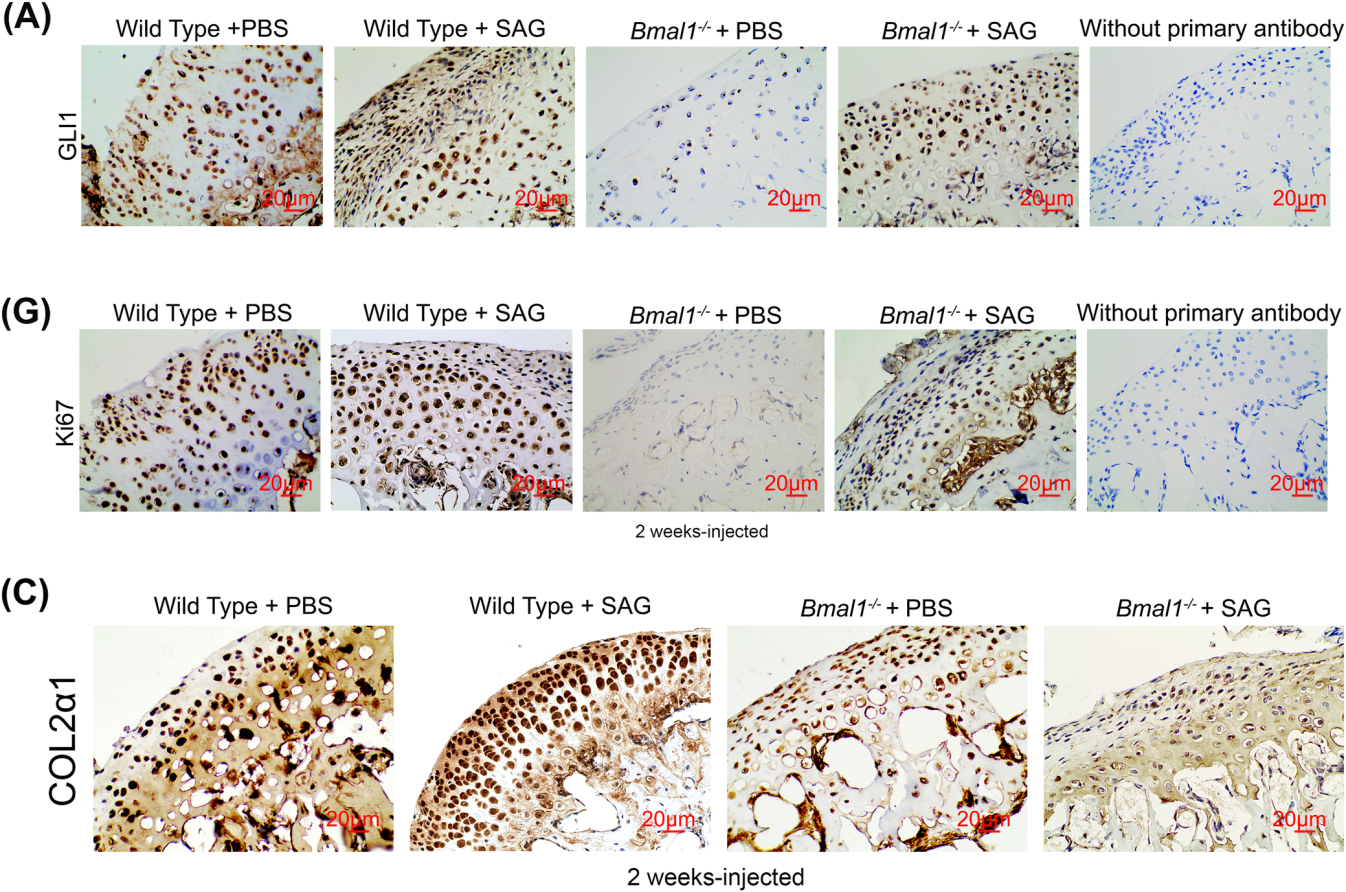

We apologize for this error.

