# Supplementary material for: Circadian BMAL1 regulates mandibular condyle development by hedgehog pathway
Source: Cell Prolif. 2019 Nov 20;53(1):e12727. doi: 10.1111/cpr.12727 (PMC6985652; doi:10.1111/cpr.12727)
Supplement: Supplementary file 1 [file CPR-53-e12727-s001.docx]

***Supplementary Figures and Table***


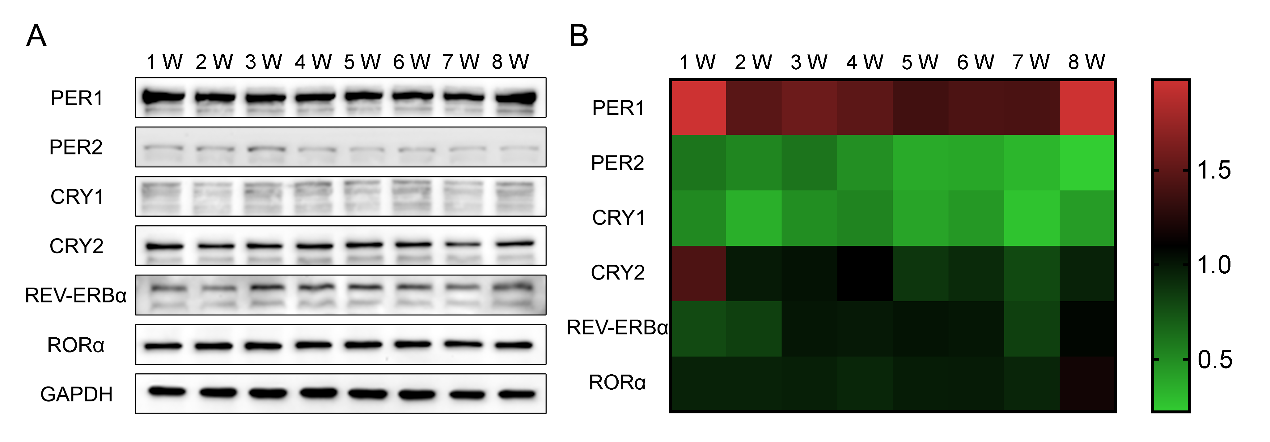


**Figure S1. Circadian regulator BMAL1 is closely correlated with the development of MCC**

(A) Western blot analysis showed the protein levels of PER1, PER2, CRY1, CRY2，REV-ERBα, and RORα in the mandibular condyle cartilages at ZT10 (n = 3 independent experiments). (B) The heatmaps shows the analysis of western blot.

**
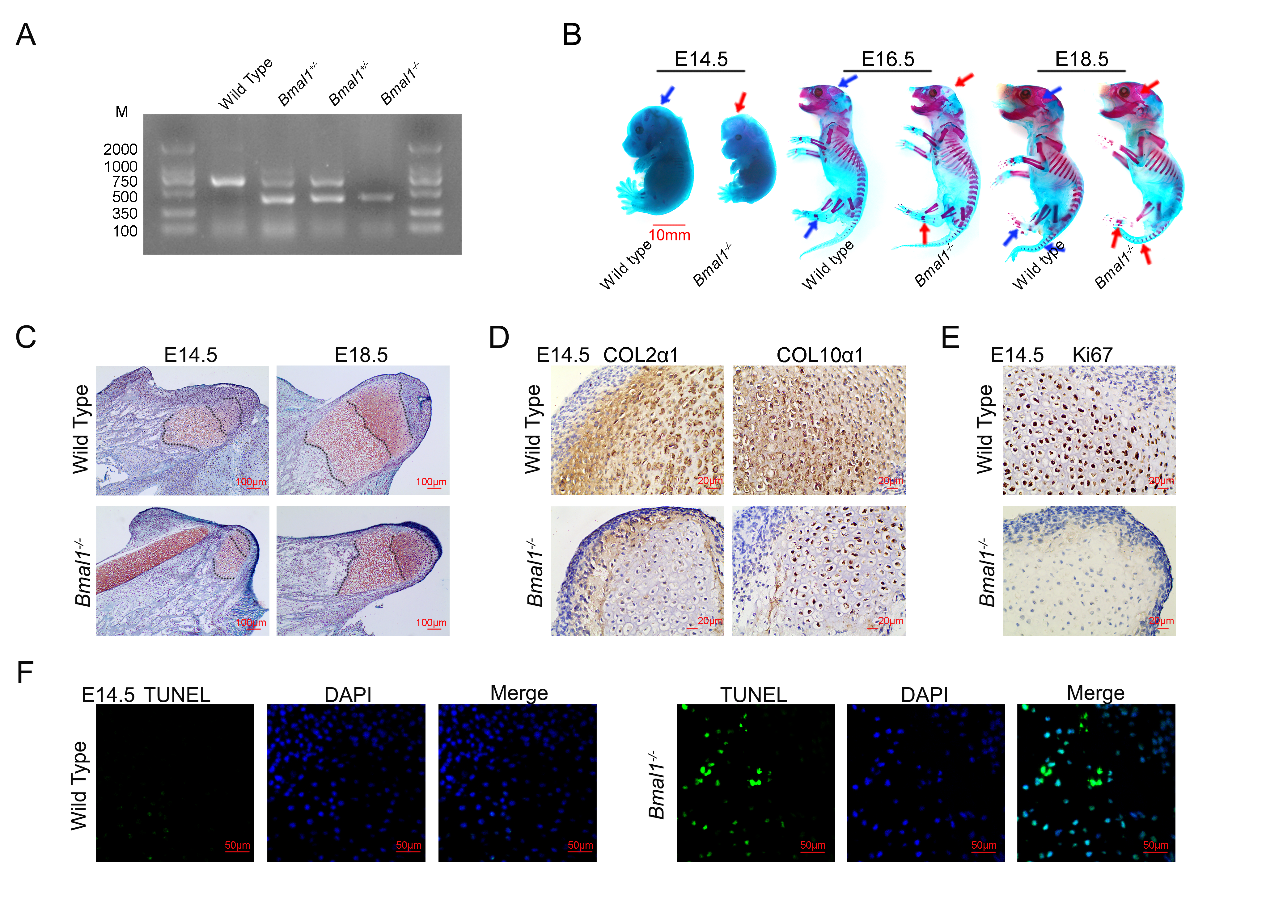
**

**Figure S2. Loss of BMAL1 delays and reduces chondrogenesis and endochondral ossification in mandibular condyle**

(A) The genotypes of WT, *Bmal1^+/-^*, and *Bmal1^-/-^* mice analysis by PCR. (B) Alcian blue and Alizarin red staining analysis of mineralized bone and cartilage from embryos at E14.5, E16.5, E18.5 (n = 3 per group). *Bmal1^-/-^* indicated by red arrows, wild-type by blue arrows. Scar bar, 10mm. (C) Representative images of S-O staining of mandibular condyles at E14.5, E18.5 (n = 3 per group). Scar bar, 100 μm. (D and E) Immunohistochemistry of COL2α1, COL10α1 and Ki67 in mandibular condyles from E14.5 embryos at ZT10 (n = 3 per group). Scar bar, 20 μm. (F) The cell apoptosis of mandibular condyles from E14.5 embryos was detected by TUNEL staining (n = 3 per group). Scar bar, 50 μm.

**
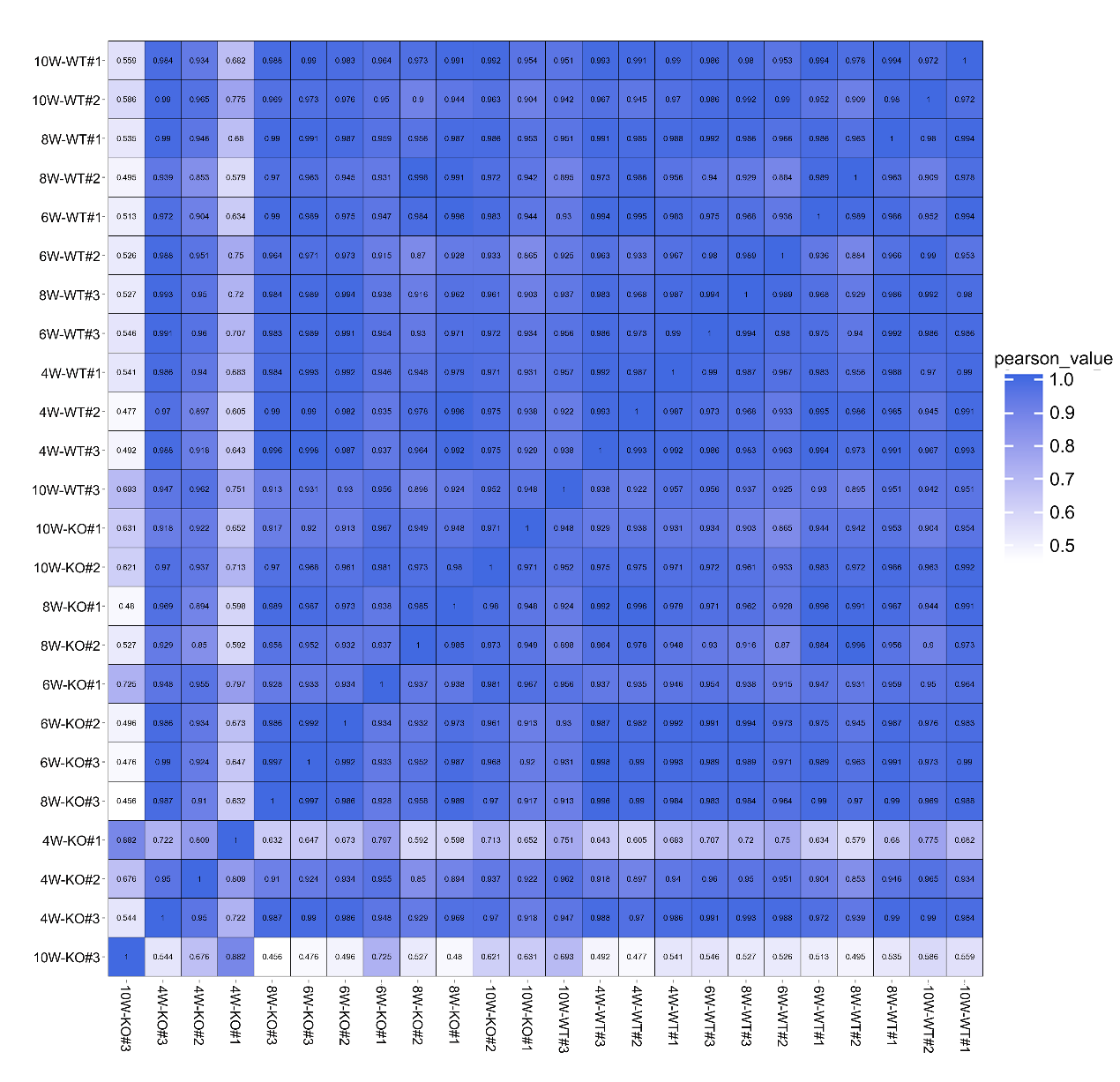
Figure S3. Correlation analysis among each sample.**

Each x- and y-axis indicates the samples and the values in each tile denoted the correlation between each sample.

**
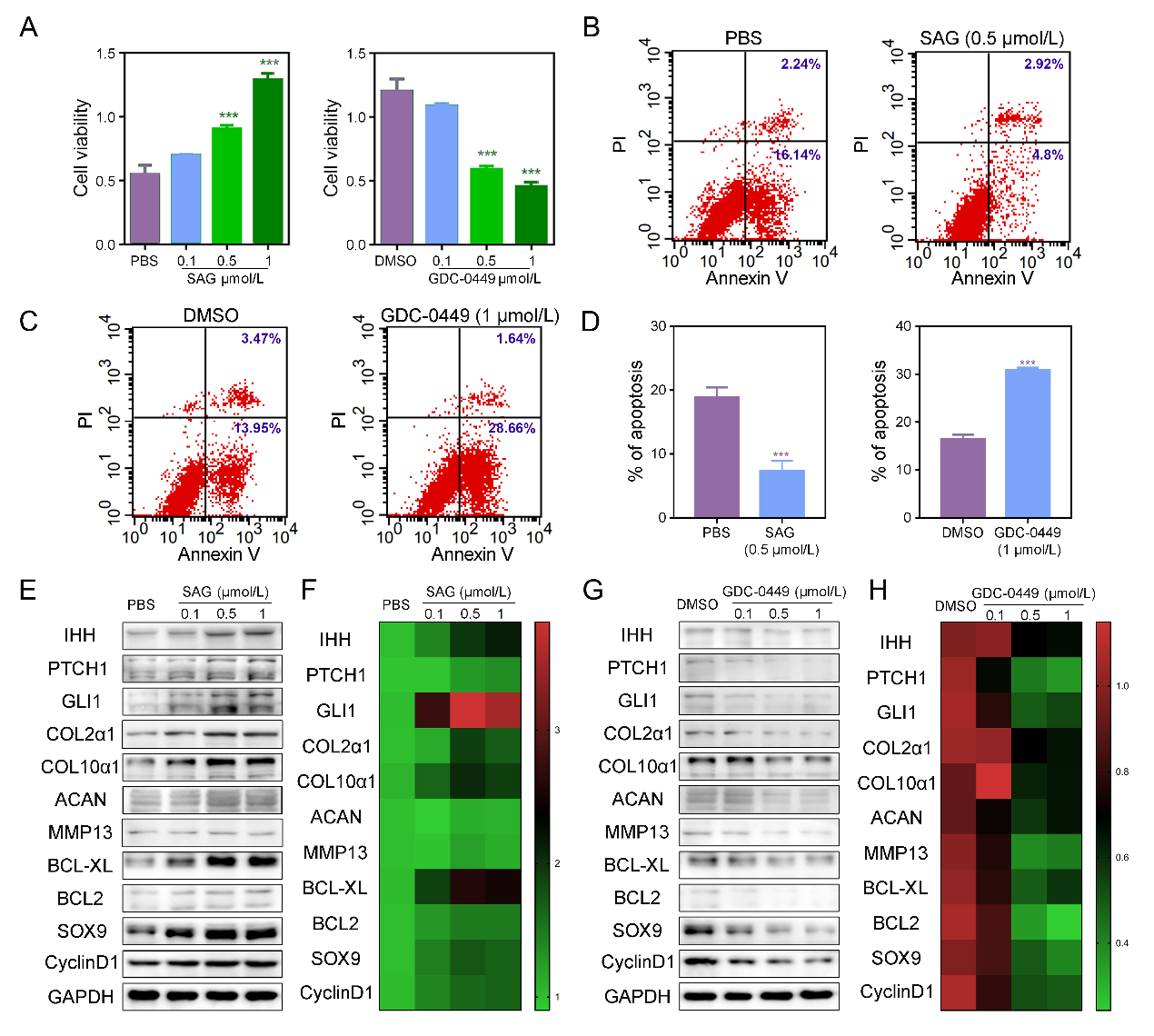
**

**Figure S4. Hh signaling is critical for endochondral ossification**

(A-C) Dose-dependent cell proliferation and apoptosis in response to Hh signaling activator SAG or inhibitor GDC-0449 in chondrocytes (n = 3 for each bar). Data represented as mean ± SEM. ^***^*P* < 0.001. (D) (D-G) Western blot analysis of Hh pathway-related proteins in chondrocytes (n = 3 independent experiments). The heatmaps (E, G) shows the analysis of western blot.


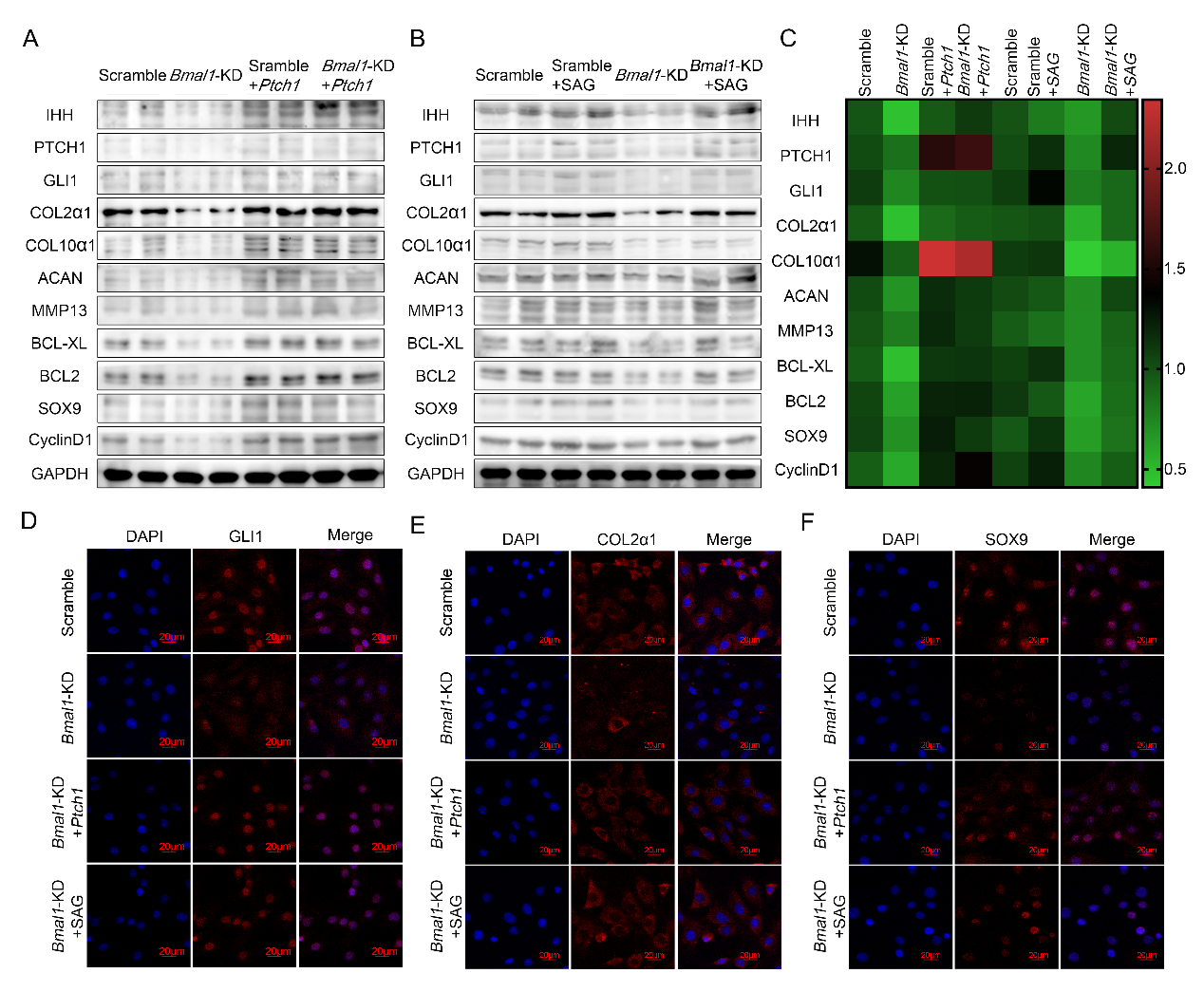


**Figure S5. BMAL1 regulates sequential chondrocyte differentiation through directly activating *Ptch1* transcription**

(A-C) Western blot analysis of Hh pathway-related proteins in BMAL1 knockdown chondrocytes with *Ptch1* overexpressing or SAG supplement (0.5 μmol/L). (n = 3 independent experiments). The heatmaps (C) shows the analysis of western blot. (D-F) Immunofluorescence of GLI1, COL2α1, SOX9 in BMAL1 knockdown chondrocytes with *Ptch1* overexpressing or SAG supplement (0.5 μmol/L). (n = 3 independent experiments). Scar bar, 20 μm.


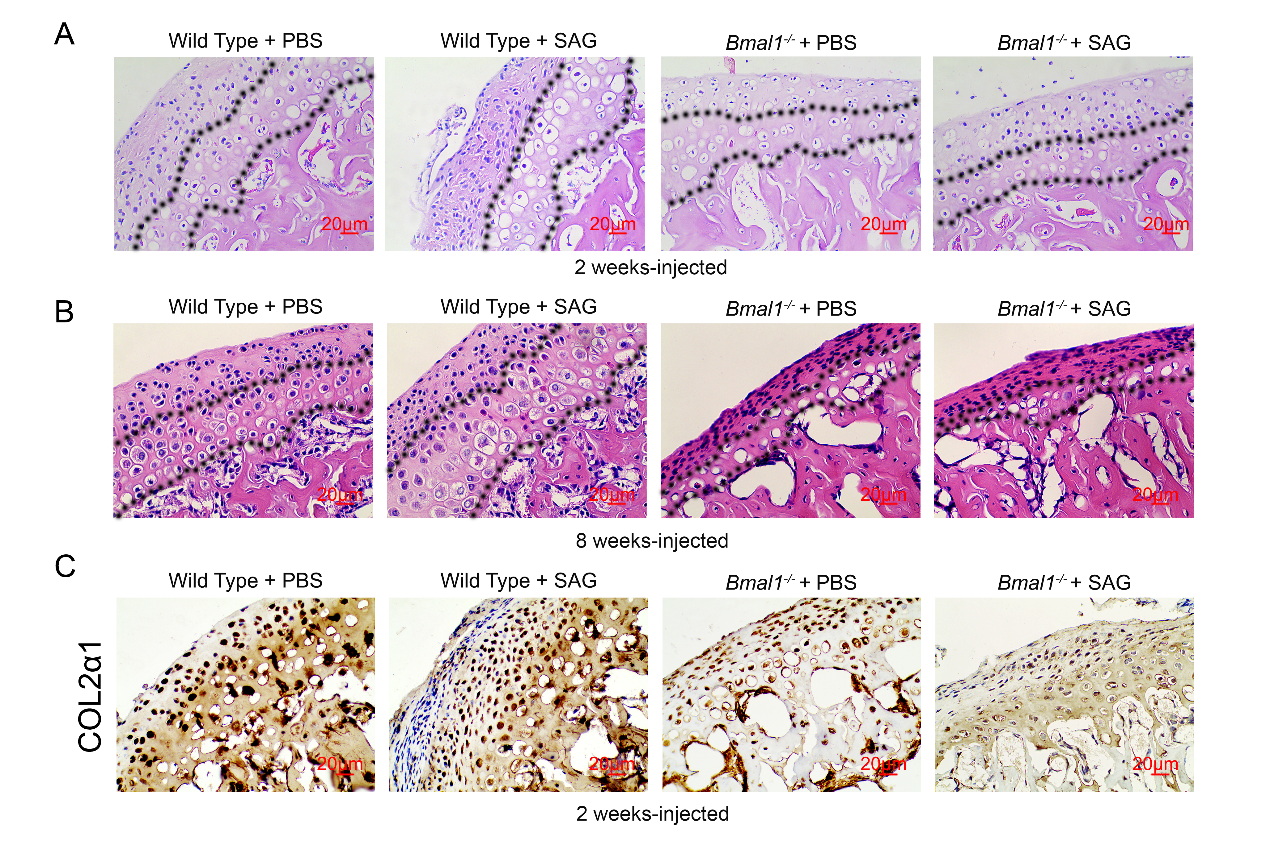


**Figure S6. Phenotypes caused by BMAL1-deficiency are rescued by Hh signaling activator during prepuberty and early puberty periods**

(A-B) H&E staining of mandibles of wild-type and *Bmal1^-/-^* mice with or without injection of SAG at 2-week or 8-week-old (n = 3 per group). Scar bar, 20 μm.

(C) Immunohistochemistry of COL2α1 in mandibular condyles of wild-type and *Bmal1^-/-^* mice with or without injection of SAG at 2-week-old at ZT10 (n = 3 per group). Scar bar, 20 μm.

Table S1. Sequences are used in this study

| **Primers** | **Sequence (forward/reverse)** | **Usage** |
| --- | --- | --- |
| *Bmal1* wild type | 5'-CCACCAAGCCCAGCAACTCA-3' 5'-ATTCGGCCCCCTATCTTCTGC-3' | gene typing |
| *Bmal1*^-/-^ | 5'-CCACCAAGCCCAGCAACTCA-3' 5'-TCGCCTTCTATGGCCTTCTTGACG-3' | gene typing |
| *Bmal1* flox | 5'-ACTGGAAGTAACTTTATCAAACTG-3' 5'-CTGACCAACTTGCTAACAATTA-3' | gene typing |
| Twist2 Cre  wild type | 5'-CGTCTCAGCTACGCCTTCTC-3' 5'-TCACGAGGAGAGATACACTGG-3' | gene typing |
| Twist2 Cre  allele | 5'-AACTTCCTCTCCCGGAGACC-3' 5'-CCGGTTATTCAACTTGCACC-3' | gene typing |
| *m-Per1* | 5'-GGTTC AGGATCCCACGAAG-3' 5'-AAGAGTCGATGCTGCCAAAG-3' | qRT-PCR |
| *m-Per2* | 5'-CACACTTGCCTCCGAAATAACTC-3' 5'-AGCGCACGGCTGTCTGA-3' | qRT-PCR |
| *m-Cry1* | 5'-CGTCTGTTTGTGATTCGGGG-3' 5'-ATTCACGCCACAGGAGTTGC-3' | qRT-PCR |
| *m-Cry2* | 5'-GTGGAGGTGGTGACTGAGAACTC-3' 5'-GGGTGGTTTCTGCCCATTC-3' | qRT-PCR |
| *m-Bmal1* | 5'-AACCTTCCCGCAGCTAACAG-3' 5'-AGTCCTCTTTGGGCCACCTT-3' | qRT-PCR |
| *m-Clock* | 5'-GGCGTTGTTGATTGGACTAGG-3' 5'-GAATGGAGTCTCCAACACCCA-3' | qRT-PCR |
| *m-Rev-erbα* | 5'-CCCTGGACTCCAATAACAACACA-3' 5'-GCCATTGGAGCTGTCACTGTAG-3' | qRT-PCR |
| *m-Gapdh* | 5'-TTCACCACCATGGAGAAGGC-3' 5'-GGCATGGACTGTGGTCATGA-3' | qRT-PCR |
| *Bmal1* | 5'-CTCCTCTGACACCGCACCCC-3' | Knockdown |
| *Ptch1* | 5'-CCCCTGGATGTCTTGGTGAC-3' 5'-TTCAGCAGGCCAGAGAAATGT-3' | Chip |
| *Ihh* | 5'-AGGAGGCAGGGACATGGATAG-3' 5'-CCGGCCGCCAATAAATAG-3' | Chip |

***Supplementary Materials and Methods***

**Chondrocyte isolation, culture,** **viral infection and treatments**

The mandibular condylar cartilages were dissected from 3-week-old rat aseptically and digested for 30 min with 0.25% proenzyme, followed by 8 h with 0.2% type II collagenase in DMEM/F12 medium (Hyclone). The released cells were resuspended and cultured in DMEM/F12 medium supplemented with 10% fetal bovine serum (Gibco), 1% penicillin (100 U/mL)/streptomycin (100 μg/mL) in a humidified incubator at 37℃, 5% CO_2_.Viral packaging was purchased from Cyagen Biosciences Inc. (Guangzhou, China). For viral inoculation, we incubated chondrocytes with the viral supernatants (MOI = 10) in DMEM/F12 supplemented with 10% FBS, at 37°C. For 12-hr we replaced with fresh complete medium. For knockdown, the CRISPR/CAS9 system with Lentiviral Vector was used. The sequences of the gRNAs used for the target genes and the primers used for amplification are listed in Supplementary Table 1. For overexpression, primary chondrocytes were transfected with Lentivirus gene expression vector (3rd generation) containing r*Arntl* (NM_024362.2) or r*Ptch1* (NM_053566.1) gene sequence. For treatments, the chondrocytes were incubated for 72 hours in the presence of GDC-0449 (dose: 0.1, 0.5, 1 μmol/L), SAG (dose: 0.1, 0.5, 1 μmol/L) or a vehicle control (DMSO or PBS ≤ 0.1%).

**Skeletal preparation (alizarin red-alcian blue staining)**

Embryos were harvested at E14.5, E16.5 and E18.5. The Embryos, after removed skin and evisercate organs, were fixed overnight in 95% ethanol and stained in Alcian Blue solution (150 µg Alcian Blue, 800 mL 98% ethanol, 200 mL acetic acid) for 2 days. After overnight in 95% ethanol, the embryos were transferred to 2% potassium hydroxide (KOH) solution for 24-hr and then stained overnight in Alizarin Red solution (50 µg Alizarin Red in 1 liter 2% KOH). Finally, embryos were washed with water and placed in 1% KOH/50% glycerol until destained, imaged and stored in 80% glycerol.

**RNA-sequencing and analysis**

Total RNA was quantified using NanoDrop 2000 (Thermos Fisher Scientific) and followed by assessment of integrity by Agilent TapeStation (Agilent) with RIN values of > 8 as passed. Sequencing libraries were constructed using TruSeq RNA Sample Prep kit (Illumina) with Poly-A pull down rRNA depletion. Finally, the libraries were sequenced on BGISEQ sequencer with a read length of 50 bp single-end reads. Raw reads above the filtering threshold (≥ 50 bp) were chosen for mapping. Sequencing primer and adapters were eliminated using Cutadapt (<https://cutadapt.readthedocs.io/en/stable/>) and the remaining reads were aligned to UCSC mm8 using bowtie^1^. Bioconductor package edgeR^2^ in R was used to identify differentially expressed genes. Gene expression was quantified using RPKM values. Gene ontology (GO) enrichment analysis and Kyoto Encyclopedia of Genes and Genomes (KEGG) enrichment analysis were performed using R package clusterProfiler^3^. Gene expression was quantified using RPKM values.

**Immunofluorescence (IF)**

Cells were seeded and cultured on sterile glass cover slips in 12-well plates. When approaching to 70%-80% confluence, cells were fixed with 4% formaldehyde, permeabilizated with 0.5% Triton X-100, and then blocked with 2% bovine serum albumin. Later, cells were stained with primary antibodies COL2α1 (abcam, ab13586, 1:100), SOX9 (abcam, ab185966, 1:50), PTCH1 (abcam, ab53715, 1:100), GLI1 (abcam, ab151796, 1:100) overnight at 4°C. For IF, cells were incubated with anti-rabbit Alexa Fluor 594 (Invitrogen) secondary antibodies (1:300) for 1-hr at room temperature, following labeling cell nucleus by DAPI. Image J was used to count the number of cells.

**EdU** **staining assay**

For *in vitro* EdU assay, the chondrocytes were cultured for 2-hr in DMEM/F12 medium containing 20 μmol/L EdU (Ribobio, Guangzhou, China). The cells were fixed and neutralized with 2 mg/mL glycine. Fixed cells were permeabilized by 0.5% Triton X-100 for 10 min, and were stained by 10 μmol/L fluorescent dye. Stained cells were analyzed by ﬂow cytometer (BD Biosciences).

**Quantitative reverse transcription (qRT)-PCR analysis**

Total RNA was extracted via Trizol (Takara, Tokyo, Japan) according to the manufacturer’s protocols. cDNA synthesis was carried out with oligo dT primers and reverse transcriptase (Takara). Real-time RT–PCR was performed using SYBR Green PCR protocol and the ABI 7300 real-time PCR system (Applied Biosystems, Carlsbad, CA, USA). Relative mRNA expression was normalized by GAPDH using 2^-△△Ct^ method. Primers used for amplification are listed in Supplementary Table 1.

**Western blot** **analysis**

Protein samples were obtained from cells or tissues by utilizing RIPA lysate and ultrasonic oscillation. After denaturation by SDS-PAGE loading buffer, protein extracts were fractionated on the SDS–polyacrylamide electrophoresis gel and transferred onto the 0.45 μm Polyvinylidene Fluoride membranes. The membrane was blocked in 5% skim milk and then probed with primary antibodies against BMAL1 (Abcam, ab93806, 1:1000), COL2α1 (Abcam, ab13586, 1:1000), SOX9 (Abcam, ab185966, 1:500), ACAN ((Proteintech, 13880-1-AP, 1:500), COL10α1 (ABclonal, A6889, 1:100), MMP13 (Proteintech l, 18165-1-AP, 1:500), PTCH1 (abcam, ab53715, 1:500), GLI1 (Abcam, ab151796, 1:500), IHH (Proteintech, 13388-1-AP, 1:500)，BCL2(Proteintech, 12789-1-AP, 1:500), BCL-Xl(Proteintech, 10783-1-AP, 1:500), GAPDH(Proteintech, 10494-1-AP,1:10000). Next day, membranes were incubated with secondary anti-rabbit antibodies (Santa Cruz Biotechnology Inc, 1:2000), followed by exposing in the appearance of the Western Blotting Detection Kit (GE Healthcare, cat#: RPN2106). For quantitative analysis, we used the Image J software to measure the gray value of each western blot lane, and divided the gray value of the target protein by the GAPDH gray value for normalization. The data of Scramble or vehicle or PBS or DMSO group was set as 1.

**Cell proliferation assay**

Cell proliferation was evaluated using Cell Counting kit-8 (CCK-8; Beyotime Institute of Biotechnology) in accordance with the manufacturer's protocol. Briefly, cells were seeded into 96-well plates at a density of 5×10^3^ cells/well and incubated for 48-hr at 37°C. Then, 10 % CCK-8 (90 µL DMEM/F12 with 10 µL CCK-8) was added to each well and followed by incubating at 37°C for 2-hr. The absorbance at a wavelength of 450 nm was measured after incubation. The cell viability was calculated as a percentage of the viable cells in the curcumin-treated group compared with the untreated control. Each experiment was repeated three times independently.

**Cell apoptotic assay**

Apoptosis analysis was performed to identify and quantify the apoptotic cells by using Annexin V-FITC/PI apoptosis detection kit (BD Biosciences, USA) followed by flow cytometry. Briefly, 1×10^6^ cells were stained by 5 µL Annexin V-FITC (BD Biosciences) for 20 min at room temperature protected from light. Then, 10 µL PI (5 µg/mL) in 1×binding buffer was added to each sample for 15 min in the dark. Subsequently, apoptosis in the cells was determined by using FCM (FacSCalibur; Becton-Dickinson, Franklin Lakes, USA) and Cell Quest software (Becton-Dickinson) to differentiate apoptotic cells (Annexin-V positive and PI-negative) from necrotic cells (Annexin-V and PI-positive).

**Histology, Immunohistochemistry, and TUNEL assays**

Mandibular condylar cartilages were isolated and fixed in 4% paraformaldehyde solution, following decalcification with 20% EDTA. Paraffin sections were baked at 65℃ for 1-hr, deparaffinized in xylene, rehydrated through ethanol series and rinsed with PBS. Hematoxylin-eosin staining (H&E) and Safranin O staining were performed on the basis of standard protocols in order to observe the growth and development status of cartilage tissues. For immunohistochemical staining (IHC), all antibodies, antigen retrieval was performed using proteinase K (100 mg/mL in PBS) for 30 min at room temperature. Endogenous peroxidase activity was blocked by 3% H_2_O_2_. Staining was performed using COL2α1 (Abcam, ab13586, 1:100), ACAN (Proteintech, 13880-1-AP, 1:50), COL10α1 (ABclonal, A6889, 1:100), Ki67 (Abcam, ab15580, 1:200), IHH (Proteintech, 13388-1-AP, 1:100), PTCH1 (abcam, ab53715, 1:100), GLI1 (abcam, ab151796, 1:100) antibodies, with a Vectastain ABC kit (Vector Laboratories, Burlingame, CA) followed by the DAB Substrate kit (Vector Laboratories) according to the manufacturer’s instructions. Apoptosis analysis was carried out on paraffin sections with a TUNEL assay kit according to the manufacturer’s instructions (Roche, Mannheim, Germany).

**Chromatin immunoprecipitation (ChIP) assay**

Chromatin immunopreciptation was performed as the manufacturer’s protocol (Millipore ChIP kit, 17-295). Briefly, 1×10^7^ cells were fixed with 37% formaldehyde, Glycine was then added to a final concentration of 0.125 mol/L to quench the reaction. Cells were washed twice with ice-cold phosphate-buffered saline, lysed and then sonicated to 100-200 bp DNA. The chromatin was then incubated overnight with BMAL1 antibody (ab93806; Abcam), followed by 2-hr incubation with Protein G Dynabeads (10003D; Invitrogen). Precipitated ChIP and input DNA were washed, reverse crosslinked and digested with proteinase K and RNase A. The DNA was then purified with spin columns and prepared to qRT-PCR amplification with the sequences (Supplementary Table 1). Quantitative PCR were performed in quadruplicate reactions using a SYBR green mix. The ChIP assays were each performed at least three independent times. ChIP real-time PCR was quantified via the fold enrichment method (antibody signal over IgG secondary antibody control) to obtain ΔCt (Ct of ChIP Ab- Ct of IgG Ab). ΔΔCt was calculated as ΔCt (Input or BMAL1 group) – ΔCt (Input group), and then the relative fold of enrichment was calculated as 2^-△△Ct^.

**Luciferase reporter assay**

The following luciferase assay experiments were performed on primary chondrocytes (1×10^5^ cells/well in 24-well plates). 375ng firefly luciferase reporter vectors and 125 ng Renilla luciferase reporter vectors pGL-3.0 basic were co-transfected into cells using Lipofectamine 3000 (Invitrogen) as instructed. After 48-hr cell were extracted. Firefly and Renilla luciferase activities were consecutively measured using dual luciferase reporter assay system (Promega). The luciferase signals were standardized according to the firefly/Renilla ratio to confirm the transcriptional activities of *Ptch1* promoters.

**References.**

1. Langmead B, Trapnell C, Pop M, Salzberg SL. Ultrafast and memory-efficient alignment of short DNA sequences to the human genome. *Genome Biol.* 2009;10(3):R25.

2. Robinson MD, McCarthy DJ, Smyth GK. edgeR: a Bioconductor package for differential expression analysis of digital gene expression data. *Bioinformatics.* 2010;26(1):139-140.

3. Yu G, Wang LG, Han Y, He QY. clusterProfiler: an R package for comparing biological themes among gene clusters. *Omics.* 2012;16(5):284-287.
